# Physiological models of body composition and human obesity

**DOI:** 10.1186/1743-7075-4-19

**Published:** 2007-09-20

**Authors:** David G Levitt, Steven B Heymsfield, Richard N Pierson, Sue A Shapses, John G Kral

**Affiliations:** 1 Department of Integrative Biology and Physiology, University of Minnesota, 321 Church St. S.E., Minneapolis, MN 55455, USA; 2 Merck & Co, 126 E. Lincoln Avenue, PO Box 2000, RY34-A238, Rahway, NJ 07065-0900, USA; 3 St. Luke's-Roosevelt Hospital, Columbia University College of Physicians and Surgeons, NY Body Composition Unit, 114^th ^Street and Amsterdam Ave, NY, NY 10025, USA; 4 Department of Nutritional Sciences, Rutgers University, 96 Lipman Drive, New Brunswick, NJ 08901, USA; 5 Department of Surgery, SUNY Downstate Medical Center, Box 40, 450 Clarkson Ave, Brooklyn, NY 11203, USA

## Abstract

**Background:**

The body mass index (BMI) is the standard parameter for predicting body fat fraction and for classifying degrees of obesity. Currently available regression equations between BMI and fat are based on 2 or 3 parameter empirical fits and have not been validated for highly obese subjects. We attempt to develop regression relations that are based on realistic models of body composition changes in obesity. These models, if valid, can then be extrapolated to the high fat fraction of the morbidly obese.

**Methods:**

The analysis was applied to 3 compartment (density and total body water) measurements of body fat. The data was collected at the New York Obesity Research Center, Body Composition Unit, as part of ongoing studies. A total of 1356 subjects were included, with a BMI range of 17 to 50 for males and 17 to 65 for females. The body composition model assumes that obese subjects can be represented by the sum of a standard lean reference subject plus an extra weight that has a constant adipose, bone and muscle fraction.

**Results:**

There is marked age and sex dependence in the relationship between BMI and fat fraction. There was no significant difference among Caucasians, Blacks and Hispanics while Asians had significantly greater fat fraction for the same BMI. A linear relationship between BMI and fat fraction provides a good description for men but overestimates the fat fraction in morbidly obese women for whom a non-linear regression should be used. New regression relations for predicting body fat just from experimental measurements of body density are described that are more accurate then those currently used. From the fits to the experimental BMI and density data, a quantitative description of the bone, adipose and muscle body composition of lean and obese subjects is derived.

**Conclusion:**

Physiologically realistic models of body composition provide both accurate regression relations and new insights about changes in body composition in obesity.

## Background

The body fat fraction is an important determinant of the pharmacokinetics of most drugs and can become the dominant factor for highly lipid soluble compounds such as the volatile anesthetics [1,2] or propofol [3]. The body mass index (BMI) has become the standard parameter used to predict body fat when all that is known is the subject's weight and height. Although the limitations of BMI as a predictor of body fat are well recognized [4], it has become widely accepted because of its simplicity. The regression relations that are currently used to predict body fat from BMI are based on simple empirical fits to the data. We describe a new set of regression relations that are based on a realistic model of body composition. An advantage of using this model relation is that it can be extrapolated to the very high fat fractions of the morbidly obese. The body fat prediction relations that are currently used have been derived from studies of normal to moderately obese subjects and their applicability to the morbidly obese (BMI > 40) (e.g. for bariatric surgery) is uncertain. The purpose of this paper is to derive regression relations that are based on a model of body composition and extend the model to the morbidly obese. The model validity and the regression parameters are then estimated by applying the model to a large experimental database.

Table 1 summarizes 4 recent regression equations that have been developed to predict body fat fraction from BMI as a function of sex, age and ethnicity. Three of the studies are limited to BMI < 36, and the fourth, which extends to BMI = 45 used the less accurate body density approach to determine body fat. In extrapolating data to BMI > 40 it is important to use a regression equation that is based on a realistic model of body composition in obese subjects. Two of the studies listed in Table 1 use a simple linear regression:

**Table 1:** Summary of recent BMI regression equations used to predict percent body fat for men and women.

Reference	Regression equation		BMI range	Method	Comment
	Men (N subjects)	Women (N subjects)			

Gallagher et. al. [5]	.516 -8.64/BMI +0.0012 age (192)	.637 -8.64/BMI +0.0012 age (225)	< 35	4 Comp.	Blacks and Asians differ
Jackson et. al. [28]	.0376 BMI -.0004BMI^2 ^-.478 (296)	.0435 BMI-.0005BMI^2 ^-.4624 (359)	< 45	Body density	Significant age and ethnic correlation
Deurenberg et. al. [19]	.01294BMI+.002 age -.193 (1976)	.01294BMI+.002 age -.08 (2516)	< 36	Variety	Ethnic differences.
Gallagher et. al. [9]	.00402BMI+.00177age-.2252 (214)	.01591BMI+.00072age-.1166 (290)	< 35	4 Comp	Blacks differ significantly

If there were ethnic differences, only the Caucasian regression equation is shown.

*Body Fat Fraction *= *a *+ *b BMI*

This is obviously not physiological since it predicts a fat fraction > 1 at large BMI. The other two relations in Table 1 are non-linear:

*Body Fat Fraction *= *a *+ *b BMI *- *c BMI*^2^

*Body Fat Fraction *= *a *- *b/BMI*

Equation 2, which is simply an empirical polynomial fit to the data, is also not physiological, going to negative values at large BMI. In contrast, eq. 3 used by Gallagher et. al. [5] is valid in the limit of large BMI and can be derived from a model of body composition changes in obesity (see below). A major advantage of using this model is that the parameters in the equation quantify these body composition changes. For example, it will be shown that these parameters provide information about the relative fractions of fat and non fat of the extra weight in obese subjects.

The different regression relations are then tested by comparing their predictions with a large database of body fat measurements based on the 3C (body density and total body water) approach. Although 3C or 4C (body density, total body water and mineral mass) provide the most accurate methods of measuring body fat, 2C estimates based just on the single measurement of body density remain the simplest and least expensive approach. Simple density measurements provide an approach to multiple measurements over short times (e.g. during weight loss after bariatric surgery [6]) when total body water measurements become problematic. Another purpose of this analysis is to use the models obtained from the BMI versus fat relations to obtain a more accurate set of 2C parameters relating body fat to experimental measurements of body density.

## Methods

### Subjects

Subjects were participants in previously reported [7-9] and ongoing clinical studies at the New York Obesity Research Center at St. Luke's-Roosevelt Hospital, New York. The subjects were all ambulatory adults, age 18 years or over, without any clinically significant diagnosed medical conditions. All participants signed an informed consent approved by the hospital's Institutional Review Board.

A total of 1356 subjects were included, with a BMI range of 17 to 50 for males and 17 to 65 for females. Table 2 summarizes the age and BMI distribution for the different ethnic groups.

**Table 2:** Age and BMI distribution for subjects in different ethnic groups.

Ethnic	Male/Female	N	Age Range; ave (SD)	BMI Range; ave (SD)
Caucasian	Male	234	18–97; 39.9 (18.3)	18.59–50.3; 25.6 (4.3)
	Female	355	18 – 90; 45.97 (18.0)	17.07–65.75; 25.05 (6.34)
Black	Male	124	20–86; 48.6 (17.0)	17.27–41.9; 26.2 (3.9)
	Female	233	18–88; 50.15 (17.5)	17.07–61.02; 29.04 (6.48)
Hispanic	Male	30	20–74; 37.5 (17.4)	20.08–47.9; 26.6 (5.4)
	Female	57	20–87; 42.18 (16.8)	18.84–57.46; 27.31 (7.70)
Asian	Male	59	28–89; 53.3 (18.1)	17.79–30.25; 23.9 (3.0)
	Female	61	26–88; 54.26 (17.5)	17.18–28.44; 21.73 (2.83)
Puerto Rican	Male	105	20–79; 47.0 (14.9)	18.41–41.7; 27.3 (4.7)
	Female	98	20–85; 46.99 (15.5)	19.8–43.5; 28.48 (5.0)

### Measurement of body fat

This analysis was limited to subjects for which both total body water and body density measurements were available so that the body fat could be estimated using the 3C model. Although this is slightly less accurate than 4C measurements that also incorporate DEXA measurements of mineral mass [10], the accuracy of DEXA is uncertain when applied to morbidly obese subjects [11-13]. The Siri 3C relation [14] was used to relate body fat fraction to the measurement of body density (den, kg/liter) and total body water weight fraction (TBW, liters/kg):

Fat Fraction=C1/den−C2TBW−C3C1=Dres/BC2=(1.0063Dres−1)/BC3=1/BB=1.1102Dres−1
 MathType@MTEF@5@5@+=feaafiart1ev1aaatCvAUfKttLearuWrP9MDH5MBPbIqV92AaeXatLxBI9gBaebbnrfifHhDYfgasaacH8akY=wiFfYdH8Gipec8Eeeu0xXdbba9frFj0=OqFfea0dXdd9vqai=hGuQ8kuc9pgc9s8qqaq=dirpe0xb9q8qiLsFr0=vr0=vr0dc8meaabaqaciaacaGaaeqabaqabeGadaaakeaafaqabeGabaaabaGaemOrayKaemyyaeMaemiDaqNaeeiiaaIaemOrayKaemOCaiNaemyyaeMaem4yamMaemiDaqNaemyAaKMaem4Ba8MaemOBa4Maeyypa0Jaem4qam0aaSbaaSqaaiabigdaXaqabaGccqGGVaWlcqWGKbazcqWGLbqzcqWGUbGBcqGHsislcqWGdbWqdaWgaaWcbaGaeGOmaidabeaakiabdsfaujabdkeacjabdEfaxjabgkHiTiabdoeadnaaBaaaleaacqaIZaWmaeqaaaGcbaqbaeqabeabaaaabaGaem4qam0aaSbaaSqaaiabigdaXaqabaGccqGH9aqpcqWGebardaWgaaWcbaGaemOCaiNaemyzauMaem4Camhabeaakiabc+caViabdkeacbqaaiabdoeadnaaBaaaleaacqaIYaGmaeqaaOGaeyypa0JaeiikaGIaeGymaeJaeiOla4IaeGimaaJaeGimaaJaeGOnayJaeG4mamJaemiraq0aaSbaaSqaaiabdkhaYjabdwgaLjabdohaZbqabaGccqGHsislcqaIXaqmcqGGPaqkcqGGVaWlcqWGcbGqaeaacqWGdbWqdaWgaaWcbaGaeG4mamdabeaakiabg2da9iabigdaXiabc+caViabdkeacbqaaiabdkeacjabg2da9iabigdaXiabc6caUiabigdaXiabigdaXiabicdaWiabikdaYiabdseaenaaBaaaleaacqWGYbGCcqWGLbqzcqWGZbWCaeqaaOGaeyOeI0IaeGymaedaaaaaaaa@8192@

where D_res _is the "residual mass density" [10]. Values of D_res _determined by Silva et. al. [10] were used (males: D_res _= 1.555; females: D_res _= 1.566). Although Silva et. al. found a significantly different value of D_res _for Causasian versus African American women, the difference was very small and only the average female value was used so that no ethnic difference in body fat calculations were introduced at this stage.

Total body water was measured by tritium dilution as previously described [9]. The tritium spaces were converted to total body water (kg) using a correction factor for non-aqueous hydrogen exchange and water density at 36°C (TBW = ^3^H_2_O × 0.95 × 0.994). The within person day-to-day coefficient of variation is 1.5%. Body density and volume were measured by underwater weighing using standard methods with a technical error of 0.0020 g/cm^3^. Residual lung volume was estimated after immersion in a sitting position by means of the closed-circuit O_2 _dilution method [9]. The body mass index (BMI) was defined as weight/height^2^, with weight in kg and height in meters.

### Ethnic dependence of body fat versus BMI relation

Comparison between different ethnic groups is complicated because body fat has a non-linear dependence on both age and BMI (see results) and the age distribution of the subjects differs significantly for the different ethnic groups. The approach we used to compare Black, Hispanic, Puerto Rican and Asians versus Caucasians was to pick an age and BMI range for the ethnic group and then choose an age and BMI range for the Caucasian group that had approximately the same average age and BMI. For most comparisons, the ethnic age range was 20 to 52 and the BMI range was 20 to 34. A smaller BMI range was used for Asians. Also, because Hispanic women had an unusual age distribution, an age range of 20 to 60 was used.

### Physiological models of body composition

Writing total body weight as the sum of that of a standard lean reference subject (W_0_) plus the additional weight of the obese subject (W_1_) with each component having fat weight F_0 _and F_1 _with fat fractions f_0 _and f_1_:

*W *= *W*_0 _+ *W*_1 _= *F*_0_/*f*_0 _+ *F*_1_/*f*_1_

W_1 _corresponds to the weight of the extra adipose tissue, plus the additional muscle and bone, etc. that is required to support this adipose tissue. Assuming that W_0 _scales as the square of the height (W_0 _= b H^2^), and solving for the body fat fraction using BMI = W/H^2 ^and defining BMI_0 _= W_0_/H^2^:

*F = F*_0 _+ *F*_1 _= *f*_0_*W*_0 _+ *f*_1_*W*_1 _= *f*_1_*W *+ *W*_0_(*f*_0 _- *f*_1_) → *Fat fraction*(*BMI*) = *F*/*W *= *f*_1 _+ (*f*_0 _- *f*_1_)*BMI*_0_/*BMI*

Two different models will be used. For model I, it is assumed that the reference state is the zero fat condition (f_0 _= 0):

*Fat fractionI*(*BMI*) = *f*_1 _- *f*_1_*BMI*_0_/*BMI*

Equation (7) is of the same form as the regression eq.(3) used by Gallagher et. al. [5] (Table 1). For Model II, the general form of eq. (6)is used, with fat fractions f_0 _and f_1_. Equation (6) describes fat fraction for the case where BMI > = BMI_0_. For Model II it will be assumed that lean subjects that have a BMI less than the model value of BMI_0 _have a constant fat fraction of f_0_:

Fat fractionII(BMI)={f1+(BMI0/BMI)[f0−f1]BMI>=BMI0f0BMI<BMI0
 MathType@MTEF@5@5@+=feaafiart1ev1aaatCvAUfKttLearuWrP9MDH5MBPbIqV92AaeXatLxBI9gBaebbnrfifHhDYfgasaacH8akY=wiFfYdH8Gipec8Eeeu0xXdbba9frFj0=OqFfea0dXdd9vqai=hGuQ8kuc9pgc9s8qqaq=dirpe0xb9q8qiLsFr0=vr0=vr0dc8meaabaqaciaacaGaaeqabaqabeGadaaakeaacqWGgbGrcqWGHbqycqWG0baDcqqGGaaicqWGMbGzcqWGYbGCcqWGHbqycqWGJbWycqWG0baDcqWGPbqAcqWGVbWBcqWGUbGBcqWGjbqscqWGjbqscqGGOaakcqWGcbGqcqWGnbqtcqWGjbqscqGGPaqkcqGH9aqpdaGabeqaauaabeqaciaaaeaacqWGMbGzdaWgaaWcbaGaeGymaedabeaakiabgUcaRiabcIcaOiabdkeacjabd2eanjabdMeajnaaBaaaleaacqaIWaamaeqaaOGaei4la8IaemOqaiKaemyta0KaemysaKKaeiykaKIaei4waSLaemOzay2aaSbaaSqaaiabicdaWaqabaGccqGHsislcqWGMbGzdaWgaaWcbaGaeGymaedabeaakiabc2faDbqaaiabdkeacjabd2eanjabdMeajjabg6da+iabg2da9iabdkeacjabd2eanjabdMeajnaaBaaaleaacqaIWaamaeqaaaGcbaGaemOzay2aaSbaaSqaaiabicdaWaqabaaakeaacqWGcbGqcqWGnbqtcqWGjbqscqGH8aapcqWGcbGqcqWGnbqtcqWGjbqsdaWgaaWcbaGaeGimaadabeaaaaaakiaawUhaaaaa@70AB@

The fat fraction for Model II (eq.(8)) depends on 3 adjustable parameters: 1) BMI_0_, the BMI of the lean reference subject; 2) f_0_, the fat fraction of the lean reference subject; and 3) f_1_, the fat fraction of the additional weight of the obese subject.

An age dependent form of Model I was also used:

*Fat fractionI*(*BMI, Age*) = *f*_1 _- *f*_1_*BMI*_0_/*BMI *+ *c Age*

### Fat fraction dependence on body density

The derivation of the relationship between body fat fraction and body density follows that of Siri [14] and Brozek et. al. [15] and uses the same model assumptions as were used above for the relationship between fat fraction and BMI. The total body weight (W) is partitioned into a reference weight W_0 _and an extra W_1 _with fat fractions f_0 _and f_1 _and density d_0 _and d_1_. Defining A = W_1_/W, the following relation for total body density (den) is obtained:

*Volume *= *W*_0_/*d*_0 _+ *W*_1_/*d*_1 _→ 1/*den *= *Volume*/*W *= (1 - *A*)/*d*_0 _+ *A*/*d*_1_

The fat fraction is described by:

*Fat fraction *= *F*/*W *= *f*_0_(1 - *A*) + *f*_1_*A*

Solving eq. (10)for A and substituting in eq.(11), one obtains the final expression relating fat fraction to body density:

Fat fraction=a/den−ba=(f1−f0)∗(d0d1)/(d0−d1)b=(d1f1−d0f0)/(d0−d1)
 MathType@MTEF@5@5@+=feaafiart1ev1aaatCvAUfKttLearuWrP9MDH5MBPbIqV92AaeXatLxBI9gBaebbnrfifHhDYfgasaacH8akY=wiFfYdH8Gipec8Eeeu0xXdbba9frFj0=OqFfea0dXdd9vqai=hGuQ8kuc9pgc9s8qqaq=dirpe0xb9q8qiLsFr0=vr0=vr0dc8meaabaqaciaacaGaaeqabaqabeGadaaakeaafaqabeGabaaabaGaemOrayKaemyyaeMaemiDaqNaeeiiaaIaemOzayMaemOCaiNaemyyaeMaem4yamMaemiDaqNaemyAaKMaem4Ba8MaemOBa4Maeyypa0JaemyyaeMaei4la8IaemizaqMaemyzauMaemOBa4MaeyOeI0IaemOyaigabaqbaeqabeGaaaqaaiabdggaHjabg2da9iabcIcaOiabdAgaMnaaBaaaleaacqaIXaqmaeqaaOGaeyOeI0IaemOzay2aaSbaaSqaaiabicdaWaqabaGccqGGPaqkcqGHxiIkcqGGOaakcqWGKbazdaWgaaWcbaGaeGimaadabeaakiabdsgaKnaaBaaaleaacqaIXaqmaeqaaOGaeiykaKIaei4la8IaeiikaGIaemizaq2aaSbaaSqaaiabicdaWaqabaGccqGHsislcqWGKbazdaWgaaWcbaGaeGymaedabeaakiabcMcaPaqaaiabdkgaIjabg2da9iabcIcaOiabdsgaKnaaBaaaleaacqaIXaqmaeqaaOGaemOzay2aaSbaaSqaaiabigdaXaqabaGccqGHsislcqWGKbazdaWgaaWcbaGaeGimaadabeaakiabdAgaMnaaBaaaleaacqaIWaamaeqaaOGaeiykaKIaei4la8IaeiikaGIaemizaq2aaSbaaSqaaiabicdaWaqabaGccqGHsislcqWGKbazdaWgaaWcbaGaeGymaedabeaakiabcMcaPaaaaaaaaa@76F1@

Siri [14] considered two different models. The first was for the case where the reference condition (W_0_) was fat free (f_0 _= 0) and the extra weight (W_1_) was pure fat (f_1 _= 1). Assuming a fat free mass density (d_0_) of 1.1 and a fat density (d_1_) of 0.9007 [16]:

*Fat fraction*(*Siri I*) = 4.971/*den *- 4.519

Siri's second model used for W_0 _a standard reference man with f_0 _= 0.14 and d_0 _= 1.063 and values for W_1 _of f_1 _= 0.62 and d_1 _= 0.948:

*Fat fraction*(*Siri II*) = 4.206/*den *- 3.187

Brozek et. al. [15] considered a large number of combinations f_0_, f_1_, d_0 _and d_1_. The most frequently referenced of these models is based on f_0 _= 0.153; f_1 _= 0.73; d_0 _= 1.064; and d_1 _= 0.938:

*Fat fraction*(*Brozak*) = 4.570/*den *- 4.142

### Statistics

All statistical significance tests represent the standard two tailed t-test with unequal variance. All of the parameter optimization was carried out using the Statistics option in Maple (Maplesoft) which minimizes the mean square residual error (MSR) (= sum(X_model_-X_exp_)^2^/N). The average error of an individual measurement is roughly equal to the square root of the MSR. Both linear and non-linear optimization was used. For optimization of the non linear equations it was essential to select initial starting values in approximately the correct range.

## Results

### Sex, age and ethnic dependence of BMI versus body fat fraction

Tables 3 and 4 list the average body fat fraction for 3 different age brackets and different BMI ranges for Caucasian males and females. Since each age bracket has nearly identical values of the average BMI, a difference in fat fraction indicates an age dependence in the BMI versus fat regression relationship.

**Table 3:** Caucasian males: Dependence of fat fraction on age for two BMI ranges.

BMI Range	Ave age (SD)	Age range	Ave BMI	Ave Fat Fraction	N
18 – 24	22.00 (2.52)	18 – 26	22.08 (1.25)	0.0927 (.048)	30
	29.03 (2.34)	26 – 33	22.23 (1.19)	0.113 (.048) (NS)	31
	52.83 (19.42)	34 – 84	22.39 (1.31)	0.150 (.059) (p < .01)	30
24 – 44	25.94 (2.66)	21 – 30	27.64 (4.00)	0.165 (.087)	47
	38.17 (5.07)	31 – 48	27.42 (3.96)	0.189 (.074) (NS)	48
	66.25 (10.69)	49 – 97	27.93 (3.41)	0.264 (.077) (p < .01)	47

The p values are for comparisons to the closest younger age group.

**Table 4:** Caucasian females: Dependence of fat fraction on age for three BMI ranges.

BMI Range	Ave age (SD)	Age range	Ave BMI	Ave Fat Fraction	N
17 – 22	24.83 (3.36)	18 – 30	19.97 (1.37)	0.196 (.045)	41
	38.04 (5.87)	30 – 49	20.60 (1.07)	0.220 (.058) (p < .05)	42
	62.98 (11.26)	49 – 89	20.55 (1.01)	0.278 (.053) (p < .01)	41
22 – 25.9	25.71 (4.46)	18 – 33	23.38 (1.14)	0.24 (.053)	41
	39.56 (5.14)	33 – 51	23.54 (1.09)	0.28 (.057) (p < .01)	41
	67.75 (10.61)	52 – 88	24.17 (1.17)	0.34 (.061) (p < .01)	40
26 – 56	34.72 (7.50)	21 – 45	32.26 (7.19)	0.39 (.076)	36
	53.75 (4.87)	45 – 61	32.06 (6.16)	.42 (.060) (NS)	36
	70.49 (6.87)	62 – 90	29.36 (2.68)	0.40 (.055) (NS)	35

The p values are for comparisons to the closest younger age group

Tables 5 and 6 compare the fat fraction versus BMI for different ethnic groups. Because the different ethnic groups have different age and BMI distributions, the Caucasian age and BMI range for each comparison was adjusted so that the average age and BMI was nearly identical for the two groups that were compared (see Methods). For both males and females, there was no significant difference in the BMI vs. fat fraction relationship among Caucasians, Hispanics and Blacks and these 3 ethnic groups were grouped together in the following analysis. For both males and females, Puerto Ricans and Asians had significantly greater body fat fraction then Caucasians for the same average BMI and age.

**Table 5:** Ethnic dependence of BMI versus fat fraction for males.

	N	Age range (ave)	BMI range (ave)	Ave Fat Fract. (SD)
Caucasian	106	28 – 54 (36.4)	20–34 (25.59)	0.165 (0.074)
Black	70	20 – 52 (36.5)	20 – 34 (26.33)	0.173 (0.075) (NS)
Puerto Rican	55	20 – 52 (37.5)	20 – 34 (26.13)	0.192 (0.066) (p < .05)

Caucasian	165	20 – 52 (31.5)	20 – 34 (25.14)	0.149 (.073)
Hispanic	24	20 – 52 (31.0)	20 – 34 (26.04)	0.169 (0.072) (NS)

Caucasian	94	28 – 54 (35.6)	20 – 30 (24.68)	0.152 (.065)
Asian	35	20 – 52 (36.6)	20 – 30 (23.97)	0.19 (.071) (p < .01)

The age range of the Caucasians was adjusted to match the age range of the comparison group. The p values are for comparisons between the ethnic group and Caucasians.

**Table 6:** Ethnic dependence of BMI versus fat fraction for females.

	N	Age range (ave)	BMI range (ave)	Ave Fat Fract. (SD)
Caucasian	130	20 – 57 (37.6)	22 – 34 (25.49)	0.303 (0.074)
Black	96	20 – 52 (37.96)	20 – 34 (26.66)	0.31 (0.07) (NS)
Hispanic	38	20 – 60 (36.7)	20– 34 (25.64)	0.302 (0.09) (NS)
Puerto Rican	40	20 – 52 (36.02)	20 – 29 (26.29)	0.33 (0.058) (p < .05)

Caucasian	158	23 – 53 (35.6)	17 – 25 (21.85)	0.239 (.061)
Asian	35	23 – 53 (36.7)	17 – 27 (21.25)	0.262 (.068) (p = 0.07)

The age range of the Caucasians was adjusted to match the age range of the comparison group. The p values are for comparisons between the ethnic group and Caucasians.

### Linear multi-regression relations for fat fraction versus age and BMI

Linear age dependent multi-regression equations were determined by least squares for the different ethnic groups:

*Fat fraction = a + b BMI + c Age*

**Figure 1:**
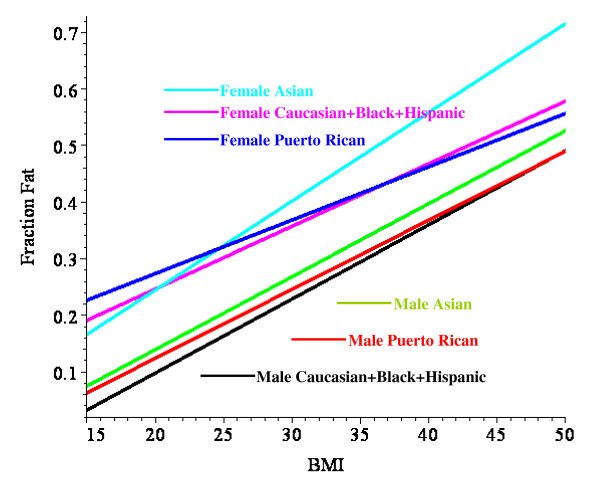
Plot of linear regression relationship (eq. (16)) between BMI and fat fraction for different male and female ethnic subject sets with the age fixed at 40 years.

Table 7 lists the regression parameters and the mean square residual error (MSR) with and without the inclusion of the age dependence for the linear fit to different ethnic subjects. The addition of the age dependence for Caucasians decreases the MSR by 35% for males and 23% for females. Combining Caucasians, Blacks and Hispanics into a single subject group does not significantly increase the MSR. These linear regression equations are plotted in Figure 1 with the age fixed at 40 years.

**Table 7:** Comparison of linear (eq. (16)) and non-linear (eq. (9)) regression expressions for predicting body fat fraction from BMI and age.

Subjects	± Age	Linear (eq. (16))				Non-linear Model I (eq. (9))			
		
		a	b	c	MSR	BMI_0_	f_1_	c	MSR
Male Caucasians	No	-.198	.0145	----	0.00426	17.98	.612	----	.00432
	Yes	-.252	.0132	.00212	0.00278	20.23	.485	.00199	.00303
Male Caucasian	No	-.177	.0137	-----	.0040	17.50	.583	----	.00407
+Hispanic+Black	Yes	-.239	.0131	.00187	0.00285	19.80	.481	.00176	.00304
Male Asian	Yes	-.188	.0129	.00173	0.00212	16.75	.423	.00173	.00224
Male Puerto Rican	Yes	-.187	.0122	.00167	0.00199	18.77	.523	.00153	.00198
Female Caucasian	No	0.0141	.0116	-----	0.00413	14.01	.732	-----	.00332
	Yes	-.0524	.0107	.00191	0.00298	15.03	.625	.00154	.00259
Female Caucasian +Hispanic+Black	No	0.0228	.0112	------	.00371	14.03	.721	-----	.00291
	Yes	-.0442	.01107	.00173	0.00275	15.00	.632	.00135	.00235
Female Asian	Yes	-.119	.0157	.00124	0.00145	12.97	.561	.00124	.00148
Female Puerto Rican	Yes	0.0463	.00943	.000972	.00164	13.38	.629	.000757	.00150

The regression parameters (either a, b and c; or BMI_0_, f_1 _and c) and the mean square residual error (MSR) for the different ethnic groups are listed.

### Linear versus non-linear model fits for fat fraction versus BMI

**Figure 2:**
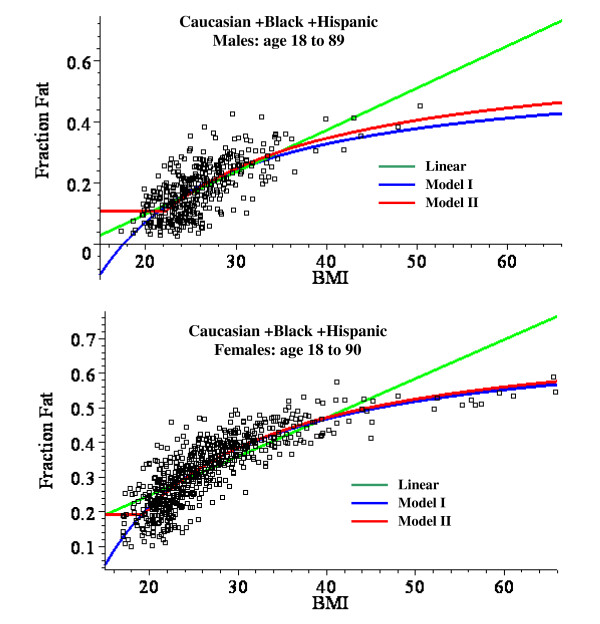
Plot of the fat fraction as a function of BMI for Caucasian + Black + Hispanic males ages 18 to 89 (top panel) and females ages 18 to 90 (bottom). The optimal least square fits to the data of the linear fit (green) and the non-linear Model I (blue) and Model II (red) are shown.

**Figure 3:**
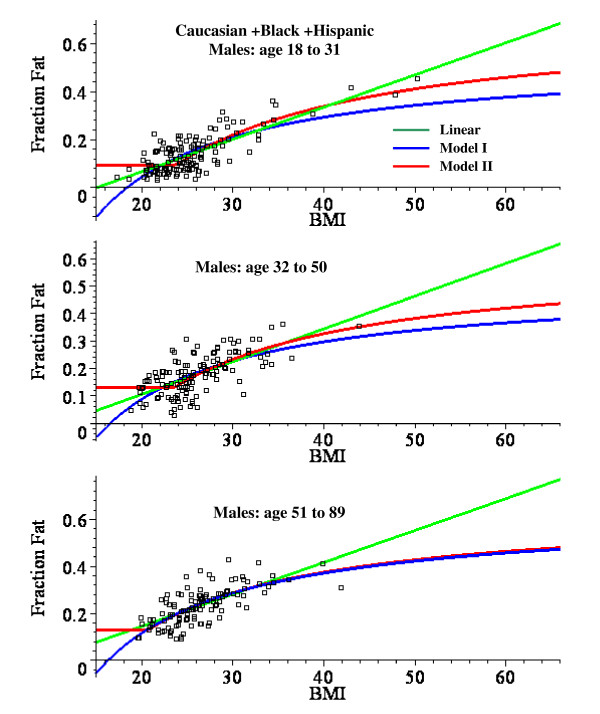
Plot of the fat fraction as a function of BMI for Caucasian + Black + Hispanic males for ages 18 to 31 (top panel), 32 to 50 (middle) and 51 to 89 (bottom). The optimal least square fits to the data of the linear fit (green) and the non-linear Model I (blue) and Model II (red) are shown.

Table 8 compares the model parameters and MSR for the Caucasian + Black + Hispanic subjects for the linear fit (eq.(1)) and for the non-linear Model I (eq.(7)) and Model II (eq.(8)) either for all ages grouped together or for three different age ranges. The non-linear model parameters (Model I: f_1 _and BMI_0_; Model II: f_0_, f_1 _and BMI_0_) were determined by a non-linear optimization routine that minimized the MSR. Figure 2 shows the plots of these optimized model fits with all ages lumped together and figs. 3 (males) and 4 (females) show similar plots for the subjects in the limited age ranges.

**Table 8:** Prediction of fat fraction from BMI for Caucasian + Black + Hispanic subjects.

Subjects	Linear eq. (1)			Model I eq. (7)			Model II eq. (8)			
	a	b	MSR	f_1_	BMI_0_	MSR	f_1_	f_0_	BMI_0_	MSR

Male: 18 – 89	-.177	.0138	.00401	.583	17.50	0.00407	.644	.109	22,24	0.00398
Male: 18 – 31	-.201	.0134	.00273	.543	19.39	0.00315	.697	.0939	23.77	0.00276
Male: 32 – 50	-.133	.0119	.00303	.505	16.54	0.00312	.606	.130	23.5	0.00297
Male: 51 – 89	-.126	.0136	.00310	.628	16.28	.00299	.638	.132	20.78	0.00298
Female: 18 – 90	+.0229	.0112	.00371	.729	14.03	.00291	.736	.193	19.44	0.00287
Female: 18 – 31	-.0347	.0119	.00252	.687	14.55	.00239	.767	.192	21.30	0.00192
Female: 32 – 50	+0.0442	.00989	.00325	.715	14.40	.00224	.730	.189	19.82	0.00221
Female: 51 – 90	+0.0803	.0104	.00237	.675	12.07	.00222	.674	.225	18.18	0.00222

Model parameters and mean square residual error (MSR) for Model I, Model II and Linear fit are listed.

**Figure 4:**
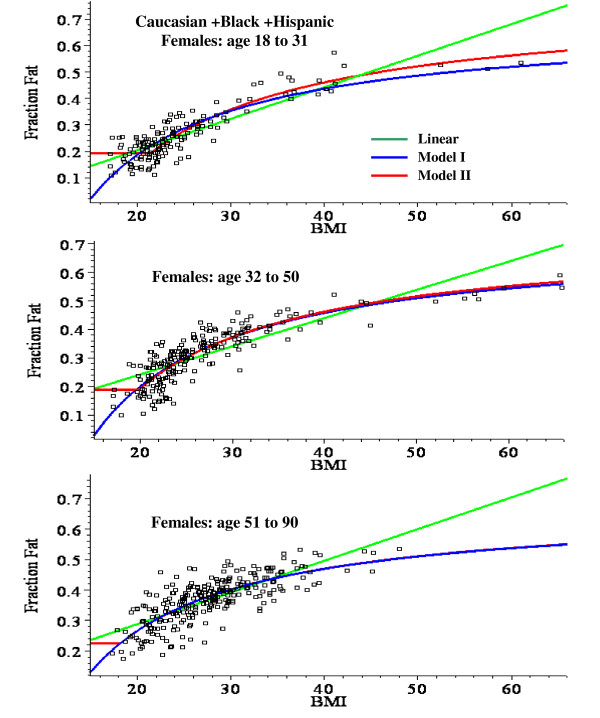
Plot of the fat fraction as a function of BMI for Caucasian + Black + Hispanic females for ages 18 to 31 (top panel), 32 to 50 (middle) and 51 to 90 (bottom). The optimal least square fits to the data of the linear fit (green) and the non-linear Model I (blue) and Model II (red) are shown.

For males, the linear plot provides a good fit to the experimental BMI data and the mean square residual error (MSR) for the non-linear models is not significantly better than the linear fit. Model II provides a significantly better fit than Model I for males, especially in the younger age range (fig. 3). For females, the linear fit significantly overestimates the value of the fat fraction for BMI values > 50 (fig. 2) and the MSR for the non-linear models is about 25% less than for the linear fit (Table 8). The Model I and II fits are nearly identical for all the female age classes (fig. 4).

A multivariable form of Model I that incorporated a linear age dependence (eq.(9)) was also fit to the male and female subjects. The optimal parameter values and the MSR for the different ethnic groups are listed on the right side of Table 7.

### Fat fraction versus density

**Figure 5:**
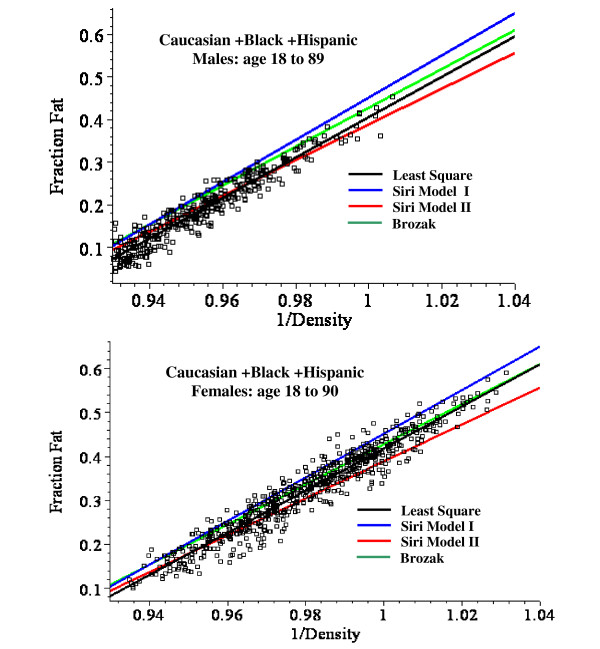
Plot of the fat fraction as a function of 1/density for males ages 18 to 89 (top panel) and females ages 18 to 90 (bottom). The black line is the optimal least square linear fit to the data. The other lines are the predictions using the Siri I (eq. (13), blue), Siri II (eq. (14), red) and Brozek (eq. (15), green) relations.

**Figure 6:**
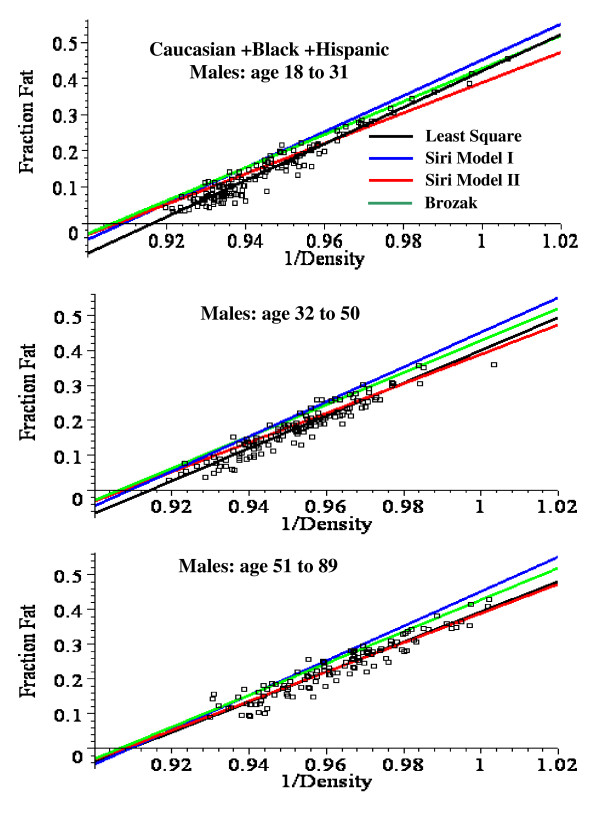
Plot of the fat fraction as a function of 1/density for Caucasian + Black + Hispanic males ages 18 to 31 (top), 32 to 50 (middle) and 51 to 89 (bottom). The black line is the optimal least square linear fit to the data. The other lines are predictions using the the Siri I (eq. (13), blue), Siri II (eq. (14), red) and Brozek (eq. (15), green) relations.

**Figure 7:**
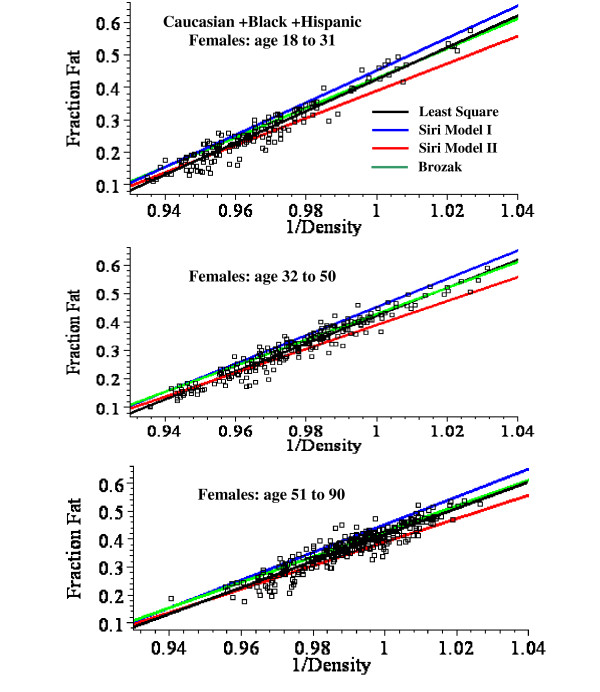
Plot of the fat fraction as a function of 1/density for Caucasian + Black + Hispanic females ages 18 to 31 (top), 32 to 50 (middle) and 51 to 90 (bottom). The black line is the optimal least square linear fit to the data. The other lines are predictions using the the Siri I (eq. (13), blue), Siri II (eq. (14), red) and Brozek (eq. (15), green) relations.

Figure 5 shows that a plot of fat fraction versus (body density)^-1 ^for Caucasian + Black + Hispanic male (top panel) or female (bottom) subjects (all ages grouped together) is accurately described by eq. (12)over the entire range of density. The different lines correspond to the different values of the linear parameters (a and b, eq.(12)). The black line represents the optimal least square set of values of a and b. Also shown are the predictions of Siri models I (eq. (13), blue line) and model II (eq.(14), red line) and of Brozek (eq. (15), green line). The least square parameters a and b (eq. (12)) are listed in Table 9. These two parameters are functions of f_0_, f_1_, d_0 _and d_1 _(eq. (12)). Using the values of f_0 _and f_1 _determined from the fit of Model II (eq. (8)) to the BMI data (Table 8), one can solve the two equations for a and b for the optimal set of values of d_0 _(density of standard lean reference subject) and d_1 _(density of extra weight in obese subjects). Listed in Table 9 are the assumed values of f_0 _and f_1 _and the corresponding values of d_0 _and d_1_. Similar plots for the age ranges of 18 to 31, 32 to 50 and 51 to 89 are shown in fig. 6 for males and in fig. 7 for females with the model parameters listed in Table 9.

**Table 9:** Prediction of fat fraction from body density for Caucasian + Black + Hispanic subjects.

Subjects	a	b	f_0_	f_1_	d_0_	d_1_	MSRls	MSRsiri1	MSRsiri2	MSRbro
Male: 18 – 89	4.751	4.345	0.109	0.644	1.0667	0.952	.000528	.00180	.000702	0.00148
Male: 18 – 31	5.046	4.625	0.094	0.65	1.0693	0.9566	.000441	.00166	.000835	0.00161
Male: 32 – 50	4.674	4.274	0.13	0.65	1.061	0.949	.000502	.00192	.000675	.00156
Male: 51 – 89	4.332	3.938	0.132	0.65	1.064	0.944	.000567	.00185	.000572	.00121
Female:18–90	4.796	4.378	0.193	0.736	1.049	0.938	.000703	.00114	.00163	.000926
Female:18–31	4.907	4.482	0.19	0.767	1.050	.935	.000677	.00101	.00132	.000935
Female:32–50	4.913	4.492	0.19	0.73	1.049	.941	.000591	.00104	.00143	.000831
Female:51–90	4.726	4.311	0.22	0.68	1.043	.947	.000793	.00129	.00196	.000999

The parameters a and b are the optimal least square values (fat fraction = a/density – b), and f_0 _and f_1 _are the fat fractions used for the determination of d_0 _and d_1 _from the values of a and b. The mean square residual error for the least square fit (MSRls), the Siri Model I (MSRsiri1, eq. (13)) and Model II (MSRsiri2, eq. (14)) and the Brozek model (MSRbro, eq. (10)) are also listed.

### Height versus weight relationship

**Figure 8:**
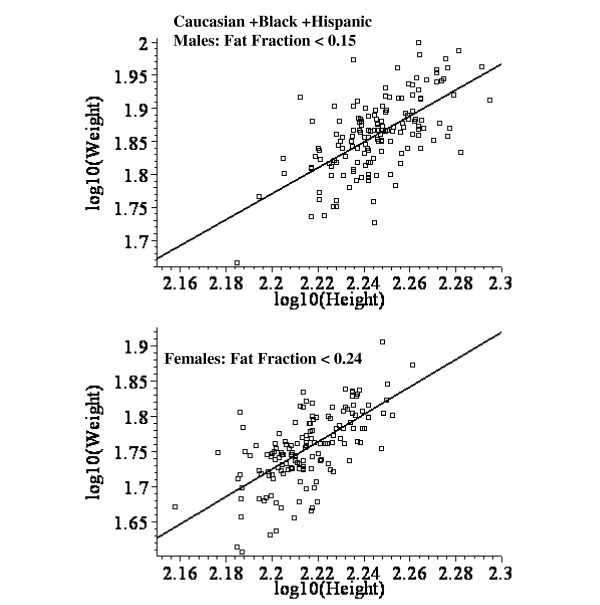
Log – log plot of height versus weight for lean Caucasian + Black + Hispanic males (top panel) and females (bottom panel). The line is the optimal least square linear fit.

In addition to the model assumptions about how the body composition changes as a function of fat content, it is also assumed that the weight of the reference subject scales as height^2 ^(W_0 _= b H^2^, see eq.(6)). Figure 8 shows a log-log plot of weight as a function of height for the Caucasian + Black + Hispanic male (top panel) and female (bottom) subjects. Since this relationship is only assumed to be valid for the lean reference condition, only lean subjects were used in these plots (males: fat fraction < 0.15; females: fat fraction < 0.24). The least square fit has a slope of 1.96 for males and 1.95 for females – confirming that W_0 _scales approximately as height^2^.

## Discussion

### Age and ethnic dependence of BMI versus fat fraction relationship

All the body fat fraction measurements used here are based on 3C model measurements of body density and total body water. This decision was made not only because the 3C model has the largest database but because DEXA measurements of bone or fat density are problematical for morbidly obese subjects [11-13]. The 3C method assumes a constant value of D_*res*_, the density of the non-fat and non-water body compartments (see eq.(4)). One can estimate the error introduced by using the 3C model from the analysis of Silva et. al. [10]. They found, for example, a value of D_*res *_of 1.555 ± 0.024 for men. Using extremes of D_*res *_of + and - one standard deviation (1.579, 1.531) yields a fat fraction range of 0.168 to 0.176 ((eq.(4)). This error is small and probably within the range of the experimental errors in body density and total body water measurement. A major assumption of this analysis is that this constant value of D_res _is valid for the morbidly obese subjects. There is no direct support for this assumption. Indirect support for this is provided by the observation that the plots of fat fraction versus either BMI using Model II (fig. 2) or density (fig. 5) do not show any obvious deviations at high fat fractions.

There is clear age dependence in the fat fraction versus BMI relation. For example, for males, the oldest age range subjects have about 60% more fat then the youngest for the same BMI (Table 3). For females the age dependence is less marked and more dependent on the BMI range. There is a roughly linear increase in fat fraction with age for women with a BMI < 26. However, for BMI in the range of 26 to 56 (average BMI of about 32), there was no significant age dependence (Table 4).

There was no significant difference in the relationship between fat fraction and BMI for Caucasians, Blacks and Hispanics for either males (Table 5) or females (Table 6) and these three ethnic groups were combined. This differs from previous studies that have found significant differences between Blacks and Caucasians [5,17-19]. In contrast, both Asians and Puerto Ricans have significantly greater fat fraction then Caucasians for the same BMI and age (Tables 5 and 6). This is a consistent observation for Asians [5,19]. The Puerto Rican result has not been previously reported.

### Regression relations for predicting fat fraction from BMI

This analysis describes a new set of regression relations based on an analysis of 1356 subjects, the largest database currently reported. A comparison of the simple linear and the non-linear physiologically based regression models are shown in figs. 2, 3 and 4. Table 8 compares the mean square residual error (MSR) of the different models. The non-linear Models I and II deviate significantly from the linear fit only for values of BMI greater than about 50. Since all the men have BMI < 51 (figs. 2 and 3), there is no clear statistical advantage of Model I or II over the linear fit for men. The linear prediction in fig. 2 overestimates the fat fraction of the two men with the largest BMI (47.9, 50.3), suggesting that the non-linear regression may be superior for morbidly obese men. However, a larger data set will be required to prove this. For the women with BMI > 50, the predictions of Model I or II are clearly superior to that of the linear fit (figs. 2 and 4) and the MSR for Model II is about 25% smaller than the MSR for the linear fit to the same data (Table 8).

Table 8 compares the linear and Model I and II fits for subjects grouped in specific age ranges. For practical prediction of fat from BMI, it is preferable to use a general multi-regression relation that directly includes the age. Table 7 lists the parameters and MSR for the age dependent forms of the linear (eq.(16)) and Model I (eq.(9)) regressions for the different ethnic groups. The addition of this linear age dependence to Model I adds another adjustable parameter that weakens the physiological interpretation of the other parameters. The parameters listed in Table 7 for the age dependent Model I should be regarded simply as a set of empirical fitting parameters. The best estimate of the actual values of the physiological parameters is provided by the data listed in Table 8 which does not include the extra age dependent parameter in the data fitting.

The age dependent linear and non-linear Model I multi-regression relations both have 3 adjustable parameters. For women, the age dependent Model I regression has an MSR 15% less than the linear model (Table 7) and this non-linear model (eq.(9)) represents the best choice for women, especially if highly obese subjects are included (see figs. 2 and 4). For men, there is no statistical difference in the accuracy of the linear and non-linear relations. However, since the non-linear relation has the correct limiting value at high fat fractions, it probably represents the best option for a general regression relation for men. In summary, this analysis suggests that the following age and BMI regression equation be used to predict body fat fraction in Caucasians, Hispanics and Blacks (see Table 7):

*Fat fraction *= *f*_1 _- *f*_1 _*BMI*_0_/*BMI *+ *c Age*

*Males*: *f*_1 _= 0.481 *BMI*_0 _= 19.8 c = 0.00176

*Females*: *f*_1 _= 0.632 *BMI*_0 _= 15.0 c = 0.00135

The corresponding regression parameters for Asians and Puerto Ricans are listed in Table 7.

The limitations of using the BMI to predict body fat fraction are well known [4] and there can be large individual variations, depending primarily on body muscle mass. The mean square residual error (MSR) values of the current analysis provide one measure of the accuracy of the fat prediction (the "average" error is equal to the square root of the MSR). For Caucasian + Hispanic + Black ethnic males the MSR for the linear age dependent prediction is 0.00285, corresponding to an average error of about 0.053 in the value of the fat fraction predicted based just on the subject's age and BMI. For females the age dependent Model I fit is better, with an MSR of 0.00235, corresponding to an average error of about 0.048 in the value of fat fraction predicted from BMI and age for the entire BMI range (17 to 65).

### Regression relations for predicting fat fraction from body density

Although the measurement of fat fraction using just the body density (2C model) is less accurate than the 3C method, it has the advantage of simplicity. It is particularly useful for following the time course of weight change (e.g. after bariatric surgery [6]) where the frequent measurements complicate body water measurements. Table 9 lists the optimal least square regression parameters (a and b, eq.(12)) for predicting fat from body density for Caucasian + Black + Hispanic males and females in different age classes. These predictions are described by the black lines in figs. 5, 6, 7.

Three different sets of regression coefficients (a and b) due to Siri [14] and Brozek et. al. [15] have been previously used to predict fat fraction from body density (eqs. (13) – (15)). Figures 5 , 6, 7 and Table 9 compare these predictions with the optimal predictions using the least square values of a and b. The MSR using these older parameters sets are from 40% to 300% larger then the MSR using the optimal values of a and b.

The Siri and Brozek relations were based on small data sets of relatively normal weight subjects. The new least square values of a and b listed in Table 9 for male and female subjects in different ages ranges should provide much more accurate relations for predicting fat fraction just from experimental measurements of body density. For men, there is a consistent age dependence in these parameters, with both a and b decreasing linearly with age. For females, the age dependence of the a and b parameters is small. The average MSR for males and females of about 0.0006 corresponds to an average error about 0.024 in the value of the fat fraction predicted using just the body density.

### Physiological models for fat fraction as a function of BMI or body density

The same model of body composition was used to derive the relationship between fat fraction versus BMI and fat fraction versus body density. The basic assumption is that, as a subject with a given height gains weight, the extra weight has a constant, fixed composition with a fat fraction f_1 _and a density d_1_. This is clearly an approximation because one would expect that in severe obesity, as the subject becomes increasingly sedentary, there should be accompanying changes in both bone and muscle mass along with other pathological changes [20]. However, the good fit of Model II for a BMI range of 17 to 65 (fig. 4) suggests that this is a roughly valid supposition.

The two models used to derive the fat fraction versus BMI differ in the body composition that is assumed for the lean "reference" subject to which the extra weight is added: for Model I, the reference subject is fat free, while, for Model II, the reference subject has a fat fraction f_0_. For young males (fig. 3), Model II is clearly superior to Model I which underestimates the fat fraction at large BMI. The difference between the two models is smaller for older males (fig.3) and females (fig. 4).

From the Model II fit to the fat fraction versus BMI data, the values of the fat fraction of the lean "reference" subject (f_0_) and of the extra weight (f_1_) can be determined. These values are listed in Table 9 for the different male and female age groups. For all males combined, the value of f_1 _is 0.644. This is nearly identical to the value of 0.64 determined by Brozek et. al. [15] from experimental weight gain or loss data in male subjects. For females the value of f_1 _is larger, about 0.73.

The model for fat fraction versus body density relates the two regression parameters (a and b) to four physiological parameters (f_0_, f_1_, d_0_, and d_1_, eq.(12)). Using the values of f_0 _and f_1 _determined from the fat fraction versus BMI regression (Table 8), these two equations for a and b can be solved for d_0 _and d_1_. Table 9 lists the assumed values of f_0 _and f_1 _and the corresponding values of d_0 _and d_1 _for the different male and female age dependent density regression relations.

With some additional assumptions, it is possible to use these 4 parameters (f_0_, f_1_, d_0_, and d_1_) to obtain a more detailed description of the tissue body composition. In Table 10, the body composition (as the fraction of total weight) is represented by muscle, bone and adipose with all the remaining tissues lumped into "other". The composition of both the lean "reference" subject and of the "extra" weight associated with the added fat are listed. It is assumed that the "other" tissue is essential functional tissue (gastrointestinal, nervous, heart, liver, etc.) that has a constant mass and, therefore, is not part of the additional "extra" weight that is added in obese subjects. It is assumed that "other" has the same weight fraction and density in the male and female "reference" subject. The experimental values of f_0_, f_1_, d_0_, and d_1 _place strong constraints on the body composition. For example, if each tissue's fat fraction and density are known then the values of f_1 _and d_1 _uniquely determine the adipose, muscle and bone composition of the "extra" tissue. Details about how the tissue weights were determined are described in the legend to Table 10.

**Table 10:** Body composition of standard male and female.

Sex	Tissue	Reference body composition			Extra body composition		
		
		Wt. Fr.	Fat Fr.	Density	Wt. Fr.	Fat. Fr.	Density
Male	Bone	0.151	0	1.4	0.0427	0	1.4
	Muscle	0.49	0	1.04	0.200	0	1.04
	Adipose	0.136	0.8	0.92	0.758	0.85	0.915
	Other	0.223	0	1.059	0	0	0
	Total	1	0.109	1.0667	1	0.644	0.952
Female	Bone	0.14	0	1.4	0.0367	0	1.4
	Muscle	0.395	0	1.04	0.0974	0	1.04
	Adipose	0.241	0.8	0.92	0.865	0.85	0.915
	Other	0.223	0	1.059	0	0	0
	Total	1	0.193	1.049	1	0.736	0.938

The body weight fraction (Wt. Fr.), the fraction of fat in each tissue (Fat Fr.) and the density of the body tissues are listed for the lean "Reference" subject and for the "Extra" weight associated with the additional fat in obese subjects. The composition is adjusted to be consistent with the experimental values of f_0_, f_1_, d_0 _and d_1 _(male: f_0 _= .109; f_1 _= .644; d_0 _= 1.0667; d_1 _= .952; female: f_0 _= .193; f_1 _= .736; d_0 _= 1.049; d_1 _= .938; see Table 8). The "Reference" adipose weight fraction = f_0_/("Reference" adipose fat fr) and the "Extra" adipose weight fraction = f_1_/("Extra" adipose fat fr). The reference bone and muscle weight fraction is derived from the values listed for the reference man [29] adjusted to give the observed male and female values of f_0_. The weight fraction and density of the "Other" tissue was assumed to be the same in males and females. The adipose density is based on a density of 0.9007 for fat and 1.00625 for the adipose non-fat. The density of "other" was adjusted to give the observed d_0_. The "Extra" bone, muscle and adipose weight fractions are uniquely determined by the values of f_1 _and d_1_.

The most uncertain value used to derive the composition in Table 10 is the value of the fat fraction of adipose tissue. A number of studies have suggested that the fat fraction of adipose tissue increases in obese subjects, possibly as a result of an increase in the individual adipose cell volume [21-23]. In Table 10 it has been assumed that adipose tissue is 80% fat in the lean references subject and 85% fat in the extra weight in obese subjects.

Table 10 provides a quantitative description of the composition of the extra weight that is gained (or lost) in obese subjects. This consists of the extra adipose tissue, plus the additional muscle and bone that is required to support this adipose tissue. In males, about 20% of this "extra" weight is muscle, while in females muscle is only about 10% of this extra weight. In both males and female, bone represents about 4% of the extra weight. This model, which uses a fixed composition of the "extra" tissue, provides a good description for the entire range of BMI (16 to 65) for females (see figs. 2 and 4). This suggests that the "extra" weight in morbidly obese subjects (BMI > 50) does not differ qualitatively from that of moderately obese subjects.

Table 10 lists the composition in terms of the weight fraction. This can be converted to absolute weight as follows. For a person with height H and weight W, the "reference" body weight = W_0 _= BMI_0 _× H^2^(eq.(6)) and the "extra" weight = W_1 _= W - W_0_, where BMI_0 _for Model II is listed in Table 8. Multiplying the "reference" weight fraction by W_0 _and the "extra" weight fraction by W_1 _gives the absolute tissue weights.

By making additional assumptions about the extracellular (ECW) and intracellular (ICW) water fraction of the different tissues ([24]), one can predict the changes in the corresponding water compartments in obesity. It is well recognized that the ratio ECW/ICW increases in obesity [7,25,26]. This is because the "extra" weight in obese subjects is primarily adipose tissue (Table 10) whose water is almost all extracellular. In a comparison of the ECW and ICW compartments in obese versus matched non-obese controls, Waki et. al [26] reported that this ratio increased from 0.63 in non-obese to 0.81 in obese females. This is similar to the predictions using the model data in Table 10 (assuming that the extracellular water weight fraction is 0.091 for muscle and 0.15 for the "extra" adipose tissue).

In addition to the model assumptions about body composition, another assumption in the derivation of eq. (6) is that the body weight of the "reference" subjects scales as height^N^, where N = 2. This is the basic assumption underlying the use of BMI as a parameter for obesity. A large, extensive review of the height versus weight relationship [27] found an average value of N of 1.92 for males and 1.45 for females. However, this review analyzed the relation between height and total body weight while eq. (6) assumes only that the "reference" weight scales as height^N^. A more relevant test is to determine the height-weight relationship for the lean subjects in this current study, assuming that these lean subjects correspond to the "reference" subjects. Figure 8 shows a log-log plot of height versus weight for lean males (fat fraction < 0.15) and females (fat fraction < 0.24). For both males and females, the average value of N is close to 2 (1.96 for males and 1.95 for females).

## Conclusion

Using a data base of 1356 subjects, new regression relations for predicting body fat fraction from either BMI or body density are derived. Although a linear regression provides a good fit to the BMI versus fat fraction for men, the linear fit significantly overestimate the fat fraction of highly obese women (BMI > 50). For women, a non-linear regression based on a physiological model of body composition provides an accurate prediction of fat fraction for the entire BMI range. In addition, regression relations for predicting fat fractions just from experimental measurements of body density are described that are much more accurate then those that are currently used. Based on the parameters obtained from the models for BMI and body density, a quantitative estimate is derived of the body composition (e.g. bone, muscle, adipose) of the standard lean "reference" subject and of the "extra" weight added in obese subjects.

## Competing interests

The author(s) declare that they have no competing interests.

## Authors' contributions

All authors have read and approved the final manuscript.

DGL performed the physiological model development, the fitting of the model parameters and the statistical analysis.

SBH made contributions to the collection of experimental data, data analysis, and critically evaluated and revised the manuscript.

RNP made significant contributions to the collection of experimental data and evaluated and revised the manuscript.

SAS made significant contributions to the collection of experimental data and evaluated and revised the manuscript.

JGK made significant contributions to the collection of experimental data and evaluated and revised the manuscript.
