# Physiological models of body composition and human obesity

**DOI:** 10.1186/1743-7075-6-7

**Published:** 2009-02-16

**Authors:** David G Levitt, Steven B Heymsfield, Richard N Pierson, Sue A Shapses, John G Kral

**Affiliations:** 1 Department of Integrative Biology and Physiology, University of Minnesota, 321 Church Street SE, Minneapolis, MN 55455, USA; 2 Merck & Co, 126 E. Lincoln Avenue, PO Box 2000, RY34-A238, Rahway, NJ 07065-0900, USA; 3 St. Luke's-Roosevelt Hospital, Columbia University College of Physicians and Surgeons, NY Body Composition Unit, 114th street and Amsterdam Avenue, NY, NY 10025, USA; 4 Department of Nutritional Sciences, Rutgers University, 96 Lipman Drive, New Brunswick, NJ 08901, USA; 5 Department of Surgery, SUNY Downstate Medical Center, Box 40, 450 Clarkson Avenue, Brooklyn, NY 11203, USA

## Abstract

Correction to Levitt DG, Heymsfield SB, Pierson Jr RN, Shapses SA, Kral JG: Physiological models of body composition and human obesity. *Nutrition & Metabolism *2007, **4**:19

## Correction

Since publication of our first article [1] we have noticed that the following corrections needed to be made. There is an error in the calculation of the body fat in the original version of this article. The tritium distribution space was not properly corrected for non-aqueous hydrogen exchange and water density resulting in estimates of percent body fat that are about 2% less then the correct percent. This produces small errors in the regression relations for the prediction of body fat from BMI or body density described originally in Tables 3, 4, 5, 6, 7, 8 and 9. The corrected tables (calculated using TBW = 3H2O × 0.96 × 0.994) are provided.

**Table 3:** Caucasian males: Dependence of fat fraction on age for two BMI ranges.

BMI Range	Ave age (SD)	Age range	Ave BMI	Ave Fat Fraction	N
18 – 24	21.86 (2.44)	18 – 25	22.19 (1.08)	0.1193 (.046)	29
	
	29.94 (2.36)	26 – 33	22.12 (1.34)	0.134 (.048) (NS)	32
	
	52.83 (19.42)	34 – 84	22.39 (1.31)	0.173 (.057) (p < .01)	30

24 – 44	25.94 (2.66)	21 – 30	27.64 (4.00)	0.188 (.084)	47
	
	38.17 (5.07)	31 – 48	27.42 (3.96)	0.211 (.072) (NS)	48
	
	66.25 (10.69)	49 – 97	27.93 (3.41)	0.284 (.075) (p < .01)	47

The p values are for comparisons to the closest younger age group.

**Table 4:** Caucasian females: Dependence of fat fraction on age for three BMI ranges.

BMI Range	Ave age (SD)	Age range	Ave BMI	Ave Fat Fraction	N
17 – 22	24.95 (3.41)	18 – 30	20.00 (1.38)	0.219 (.045)	42
	
	38.04 (5.87)	30 – 49	20.60 (1.07)	0.241 (.056) (p < .05)	42
	
	63.32 (11.18)	49 – 89	20.55 (1.01)	0.298 (.053) (p < .01)	40

22 – 25.9	26.14 (4.72)	18 – 33	23.30 (1.03)	0.26 (.049)	43
	
	39.12 (4.91)	33 – 51	23.45 (1.05)	0.30 (.055) (p < .01)	41
	
	68.12 (10.47)	52 – 88	24.12 (1.15)	0.36 (.059) (p < .01)	39

26 – 56	34.94 (6.198)	21 – 45	31.19 (6.12)	0.408 (.074)	36
	
	54.0 (4.69)	46 – 61	31.72 (5.89)	.428 (.056) (NS)	35
	
	70.49 (6.87)	62 – 90	29.36 (2.68)	0.414 (.053) (NS)	35

The p values are for comparisons to the closest younger age group

**Table 5:** Ethnic dependence of BMI versus fat fraction for males.

	N	Age range (ave)	BMI range (ave)	Ave Fat Fract. (SD)
Caucasian	129	20 – 57 (37.4)	22 – 34 (25.42)	0.321 (0.071)

Black	95	20 – 52 (37.8)	20 – 34 (26.57)	0.328 (0.074) (NS)

Hispanic	37	20 – 60 (36.1)	20 – 34 (25.40)	0.311 (0.09) (NS)

Puerto Rican	41	20 – 52 (35.7)	20 – 30 (26.18)	0.348 (0.058) (p < .05)

Caucasian	153	23 – 53 (35.41)	17 – 25 (21.72)	0.257 (.061)

Asian	35	23 – 53 (36.7)	17 – 28 (21.25)	0.282 (.066) (p = 0.07)

The age range of the Caucasians was adjusted to match the age range of the comparison group. The p values are for comparisons between the ethnic group and Caucasians.

**Table 6:** Ethnic dependence of BMI versus fat fraction for females.

	N	Age range (ave)	BMI range (ave)	Ave Fat Fract. (SD)
Caucasian	129	20 – 57 (37.4)	22 – 34 (25.42)	0.321 (0.071)

Black	95	20 – 52 (37.8)	20 – 34 (26.57)	0.328 (0.074) (NS)

Hispanic	37	20 – 60 (36.1)	20 – 34 (25.40)	0.311 (0.09) (NS)

Puerto Rican	41	20 – 52 (35.7)	20 – 30 (26.18)	0.348 (0.058) (p < .05)

Caucasian	153	23 – 53 (35.41)	17 – 25 (21.72)	0.257 (.061)

Asian	35	23 – 53 (36.7)	17 – 28 (21.25)	0.282 (.066) (p = 0.07)

The age range of the Caucasians was adjusted to match the age range of the comparison group. The p values are for comparisons between the ethnic group and Caucasians.

**Table 7:** Comparison of linear (eq. (16)) and non-linear (eq. (9)) regression expressions for predicting body fat fraction from BMI and age.

Subjects	± Age	Linear				Non-linear Model I			
		
		a	b	c	MSR	BMI_0_	f_1_	c	MSR
Male Caucasians	No	-.166	.0141	----	0.00404	17.20	.624	----	.00409
	
	Yes	-.218	.0129	.00207	0.00263	19.15	.500	.00194	.00287

Male Caucasian +Hispanic+Black	No	-.145	.0134	-----	.00380	16.71	.594	----	.00385
	
	Yes	-.206	.0127	.00182	0.00270	18.73	.496	.00172	.00288

Male Asian	Yes	-.156	.0126	.00169	0.00201	15.72	.438	.00169	.00212

Male Puerto Rican	Yes	-.155	.0119	.00163	0.00189	17.84	.536	.00150	.00188

Female Caucasian	No	0.0409	.0113	-----	0.00391	13.50	.739	-----	.00314
	
	Yes	-.0240	.0104	.00186	0.00281	14.39	.635	.00151	.00244

Female Caucasian +Hispanic+Black	No	0.0494	.0109	------	.00351	13.50	.728	-----	.00276
	
	Yes	-.0160	.0104	.00169	0.00260	14.37	.642	.00132	.00222

Female Asian	Yes	-.0903	.0153	.00122	0.00137	12.38	.573	.00122	.00140

Female Puerto Rican	Yes	0.0718	.00919	.000947	.00159	12.82	.639	.000737	.00142

The regression parameters (either a, b and c; or BMI0, f1 and c) and the mean square residual error (MSR) for the different ethnic groups are listed.

**Table 8:** Prediction of fat fraction from BMI for Caucasian + Black + Hispanic subjects.

Subjects	Linear			Model I			Model II			
	a	b	MSR	f_1_	BMI_0_	MSR	f_1_	f_0_	BMI_0_	MSR

Male: 18 – 89	-.145	.0134	.0038	.594	16.71	0.00385	.647	.129	22.00	0.00377

Male: 18 – 31	-.201	.0134	.00273	.543	19.39	0.00315	.706	.118	23.78	0.00261

Male: 32 – 50	-.133	.0119	.00303	.505	16.54	0.00312	.619	.153	23.54	0.00281

Male: 51 – 89	-.126	.0136	.00310	.628	16.28	.00299	.661	.167	21.43	0.00283

Female: 18 – 90	+.0494	.0109	.00351	.728	13.50	.00276	.745	.220	19.65	0.00272

Female: 18 – 31	-.00685	.0116	.00237	.695	13.99	.00225	.774	.214	21.30	0.00181

Female: 32 – 50	+0.0700	.00963	.00306	.723	13.86	.00212	.737	.208	19.71	0.00209

Female: 51 – 90	+0.106	.0101	.00225	.681	11.57	.00210	.682	.249	18.28	0.00210

Model parameters and mean square residual error (MSR) for Model I, Model II and Linear fit are listed.

**Table 9:** Prediction of fat fraction from body density for Caucasian + Black + Hispanic subjects.

Subjects	a	b	f_0_	f_1_	d_0_	d_1_	MSRls	MSRsiri1	MSRsiri2	MSRbro
Male: 18 – 89	4.63	4.208	0.129	0.647	1.0678	0.954	.000481	.000693	.000711	0.000553

Male: 18 – 31	4.912	4.475	0.118	0.706	1.0695	0.948	.000402	.000536	.000597	0.000532

Male: 32 – 50	4.559	4.141	0.153	0.619	1.061	0.958	.000457	.000723	.000614	.000562

Male: 51 – 89	4.231	3.821	0.167	0.661	1.0612	0.944	.000516	.000853	.000957	.000568

Female: 18 – 90	4.673	4.239	0.220	0.745	1.048	0.9376	.000640	.000813	.00202	.000662

Female: 18 – 31	4.779	4.339	0.214	0.774	1.050	.935	.000616	.00066	.00178	.000661

Female: 32 – 50	4.785	4.347	0.208	0.737	1.050	.941	.000538	.000653	.00191	.000576

Female: 51 – 90	4.606	4.175	0.249	0.682	1.041	.948	.000722	.00102	.00223	.000732

The parameters a and b are the optimal least square values (fat fraction = a/density – b), and f0 and f1 are the fat fractions used for the determination of d0 and d1 from the values of a and b. The mean square residual error for the least square fit (MSRls), the Siri Model I (MSRsiri1, eq. (13)) and Model II (MSRsiri2, eq. (14)) and the Brozek model (MSRbro, eq. (10)) are also listed.
